# The Use of Mycoendophyte-Based Bioformulations to Control Apple Diseases: Toward an Organic Apple Production System in the Aurès (Algeria)

**DOI:** 10.3390/plants11233405

**Published:** 2022-12-06

**Authors:** Oussama A. Bensaci, Toufik Aliat, Rafik Berdja, Anna V. Popkova, Dmitry E. Kucher, Regina R. Gurina, Nazih Y. Rebouh

**Affiliations:** 1 Laboratory of Improvement of the Phytosanitary Protection Techniques in Mountainous Agrosystems (LATPPAM), Agronomy Department, Institute of veterinary and Agricultural Sciences, Batna 1 University, Batna 05000, Algeria; 2 Higher National School of Forests, Khenchela 40000, Algeria; 3 Department of Environmental Management, Peoples’ Friendship University of Russia (RUDN University), 6 Miklukho-Maklaya Street, 117198 Moscow, Russia

**Keywords:** *Malus domestica*, fungal endophytes, invert emulsion, biogel, biological control

## Abstract

The present study aims to investigate the effectiveness of bioformulations based on endophytic fungi to control apple scab and Valsa canker disease in two orchards in the Aurès region (Algeria). In both orchards, the results showed that the treatment of senescent apple leaves by invert emulsions containing *Trichoderma longibrachiatum* and *Chaetomium globosum* harmed the ascogenesis of winter forms of *Venturia inaequalis* by reducing the number of ascospore-ejecting asci, the number of morphologically mature asci, and a considerable increase in the immature asci number. This antifungal activity was more essential in soil-incorporated leaves, showing the importance of the combination of treatments with cultural practices to efficiently control the apple scab disease. Furthermore, the disease incidence decreased by 52.63% and 50.68% in R’haouat and Bouhmama orchards, respectively. Moreover, the treatment of *Valsa ceratosperma* cankers with a biogel containing the endophytic yeast *Metschnikowia* sp. led to wound healing varying from 43.52% and 87.97% after 120 days but remained more considerable than conventional treatment with Folicur (tebuconazol). The current results open real opportunities concerning the implementation of eco-friendly and potent apple protection systems.

## 1. Introduction

Apple production is an integral part of the arboriculture sector, constantly improving in terms of production techniques [1,2]. This progress is justified because consumer requirements necessitate a product of high and regular yield, considerable commercial quality, and plasticity regarding cultivation techniques and because the search for new resistant genotypes against notable pests and diseases is required [3].

In 2020, global apple production reached approximately 86.44 million tonnes with a yield of 187.01 cwt/ha [4]. Algeria is a Mediterranean country whose climatic and edaphic conditions in some Northern regions of the country offer a suitable environment for apple cultivation. National apple production reached 566,824 tonnes in 2020 with a yield of 171.63 cwt/ha [4]. This yield is low compared to the global average, attributed to several climatic or agro-technical factors. On the other hand, harmful organisms are frequently encountered on apple trees and are involved in the reduction in production, causing significant damage to production quality [5]. In the Aurès province, apple production was mainly practiced in mountainous areas. The cultivars are mainly Golden Delicious (65%), Starkrimsson (16%), Royal Gala (14%), and Anna (5%), while the area planted by this fruit species increased from 3009.5 ha in 2012 to 3690 ha in 2019 where the average yield was of the order of 243.902 cwt/ha (Batna Agricultural Services Administration).

The main fungal diseases encountered in the Aurès apple orchards are the scab caused by *Venturia inaequalis* (Cooke G. Winter). The causal agent has a life cycle consisting of two phases; the saprophytic phase that occurs in the litter and the superficial layers of the soil, but this fungus is most often preserved as an overwintering immature pseudothecia [6,7,8], while the parasitic phase occupies a large part of the vegetative and reproductive growing season of the host plant [9]. In spring, from the leaf litter, projections of ascospores, which cause primary infections, occur during each rainy event; they are at the origin of primary infections [7]. After leaf penetration using ascospore arising germ tubes, the growth of *V. inaequalis* become restricted to the subcuticular level as a multi-layered and compacted mycelial structures known as stromata from which it derives its nutrients until an advanced stage of the disease. The symptoms are visible especially on leaves and fruits by the appearance of initially green, oily spots that turn brown–grey–black. These spots enlarge and may induce cracks that may in turn cause the fruit to dry out. The efficient secondary dissemination of the disease is ensured by conidia resulting from cumulative asexual reproduction during the host’s growing season [10].

Scab infection causes fruit deformation and premature leaf and fruit drop, which increase the sensitivity of the host plant to biotic and abiotic stresses, therefore, crop losses can reach 70% and the fruit can become unmarketed [11]. The mechanisms of scab resistance have been the subject of several studies and reviews [12,13,14,15,16] although most apple cultivars are susceptible to scabs including those known for their high commercial value.

Valsa cankers, also known as Cytospora canker or Leucostomial canker, are induced by the species *Valsa mali* (Miyabe and G. Yamada) [17]. It is also induced by a second species, *Valsa ceratosperma* (Tode Maire). The symptoms are characterized by the development of more or less localized cankers on the bark that enlarge and can completely surround the twigs, leading to the death of the latter, the twigs, and the entire tree [18]. Valsa canker is a devastating disease on apples that induces serious economic losses worldwide [19,20].

The occurrence of canker diseases induced by a *Valsa* complex has recently reached apple orchards in the Aurès that have faced climatic accidents such as hail or alternating cold and hot-dry periods, leading to bursting stems. Apple tree infection is carried out through pruning wounds and fruit scars [21]. Canker develops more rapidly between spring and early summer [21]. While considered a synonym to *V. mali* [22], *V. ceratosperma* (*Cytospora ceratosperma* (Tode G.C. Adams and Rossman)) is currently reclassified as a separate species [23,24,25,26,27,28]. *V. ceratosperma* is characterized by its spore heterogeneity and polymorphism and its broad host spectrum than *V. mali* [22,29,30], while rDNA-ITS sequence analysis revealed that this fungus represents heterogeneous species complex [26].

Farmers regularly follow a chemical control approach, repeatedly using a limited number of fungicides, leading to a loss of efficiency in particular given that some producers do not control phytosanitary treatment techniques. This situation has reinforced the arguments for healthy production. However, the technical specificities of the apple production system suggest the development of new approaches, especially the use of protection introns, while preserving the quantitative and qualitative status of the harvest.

Because of its economic potential and added value, apple production is more predisposed to the establishment of a biological control strategy that can ensure the attaining of a healthy and, therefore, more attractive fruit product within the markets [31,32]. The biological control of apple scab and Valsa canker using endophytic microorganisms has been subject to several studies [33,34,35,36,37,38] revealing several mechanisms of action such as antibiosis, mycoparasitism, and induced resistance [34,38,39]. The phytosanitary products of biological origin must be formulated in order to ensure its effectiveness and stability with regard to the conditions of the natural environment. In this context, we have carried out a series of experiments in two orchards in the Aurès region to test the effectiveness of biological treatments based on mycoendophyte formulations against apple scab and Valsa canker disease.

## 2. Results

### 2.1. Apple Scab Incidence after Leaf Treatment

**Figure 1:**
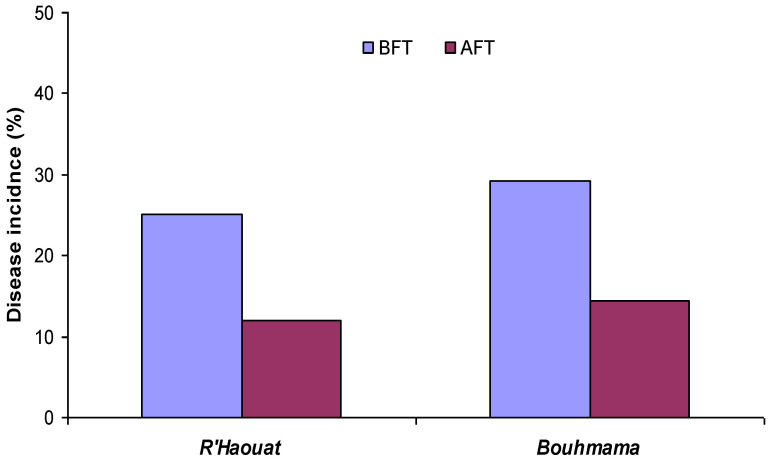
Apple scab incidence before (BFR) and after (AFT) leaf litter treatment by invert emulsions.

The incidence of scabs decreased over the year of treatment. The disease incidence decreased by 52.63% and 50.68% in R’haouat and Bouhmama orchards, respectively (Figure 1). The determination of the incidence was determined during the same period as the previous year. This indicates that litter treatments with inverted emulsions resulted in a reduction in the infectivity of the inoculum.

### 2.2. Effect of Invert Emulsion Application on Wintering Forms of V. inaequalis

After the incubation period, the reproductive structures representing the different stages of maturity of *V. inaequalis* pseudothecia were inspected in the laboratory.

For unincorporated leaves, the treatments led to a decrease in the average number of asci-ejecting ascospores (AA) compared to untreated leaves. This decrease varies depending on the fungal species used for emulsion-treated leaves containing *T. longibrachiatum* and *C. globosum* in the R’Haouat and Bouhmama orchards (*p* < 0.0001) (Table 1). This decrease is more considerable in incorporated leaves for both orchards (Table 1).

In contrast, the average number of immature asci (IA) was increased considerably in unincorporated leaves, especially in those treated with *C. globosum* emulsion in R’Haouat (Pr < 0.0001). While in the Bouhmama orchard, the reduction was 66.36% and 79.61% after treatment with *T. longibrachiatum* and *C. globosum* emulsions, respectively. In addition, the treatment of incorporated leaves led to an increase in IA by 125.85% (*T. longibrachiatum* emulsion) and by 186.49% (*C. globosum* emulsion) in the R’Haouat orchard (Pr < 0.0001) and by 164.17% (*T. longibrachiatum* emulsion) and 232.18% (*C. globosum* emulsion) in the Bouhmama orchard (Pr < 0.0001) (Table 1).

Finally, the mean number of morphologically mature asci (MA) from unincorporated leaves decreased for those treated with *T. longibrachiatum* and *C. globosum* emulsion (Pr < 0.0001) (Table 1). Whereas in the incorporated leaves, this reduction recorded average values of 43.81% (*T. longibrachiatum* emulsion) and 69.87% (*C. globosum* emulsion) for the R’Haouat orchard (Pr < 0.0001) and only 9.57% (*T. longibrachiatum* emulsion) and 69.76% (*C. globosum* emulsion) for the Bouhmama orchard (Table 1).

### 2.3. Effect of Biogel Application on Apple Canker

**Figure 2:**
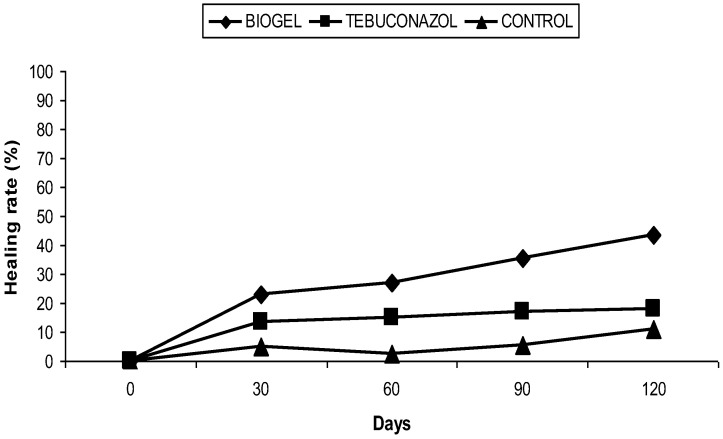
Effect of biogel application on apple canker in the R’Haouat orchard.

After the treatment of apple branches by the biogel, we noticed a gradual reduction in cankers during the follow-up period (120 days). At the R’Haouat orchard, the mean area of the canker was reduced, resulting in a healing rate of 43.52% (Figure 1), while for the branches treated with tebuconazol, the mean area of the canker was decreased, with a healing rate of 18.18%. Finally, less important healing (11.11%) was also recorded for untreated trees (Figure 2).

**Figure 3:**
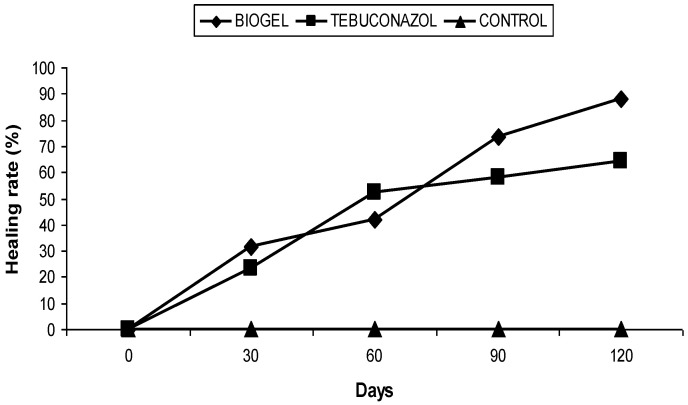
Effect of biogel application on apple canker in Bouhmama orchard.

In the second orchard (Bouhmama), the biogel treatment also led to a decrease in the average area of canker, i.e., a healing rate of 87.97% (Figure 3), whereas for the trees treated with tebuconazol, the area of cankers was reduced, resulting in a healing rate of 64.10% (Figure 3). For the untreated trees, the mean area of the cankers was increased from day 0 to day 30, stabilized on days 60 and 90, and reached 188.75 mm^2^ on the 120th day. Thus, no healing was recorded during this period.

## 3. Discussion

The biological control of apple scab disease using fungal antagonists or mycoparasites, including *Trichoderma* spp. *C. globosum*, was the object of several works [40,41,42]. In this study, the ascocarp maturity of *V. inaequalis* is closely related to the physical and chemical conditions that mark the apple litter from the late fall to early spring season [43]. The period during which the pseudothecia undergo a sequence of maturity stages of asci and ascospores were described by Meszka (2015) [44].

We have demonstrated that the treatment of apple senescent leaves with inverted emulsions leads to an alteration in the ascogenic potential and maturity of *V. inaequalis* ascocarps, considerably reducing the primary inoculum of the pathogen.

The cumulative evolution of the immature asci numbers (IA) and morphologically mature asci (MA) is probably the result of antibiotic activity of the tested endophytes that inhibit or slow down the growth and maturity of these reproductive structures, which multiplies the risks of a fatal exposure of juvenile fruiting bodies to adverse environmental conditions such as the acceleration of leaf biodegradation and, therefore, the suppression of leaf protection that wintering ascocarps benefit from. This maturity slowdown can lead to a delay or even an exclusion from the primary infection, which can be exceeded chronologically when the conditions become unfavorable.

*T. longibrachiatum* is known for its ability to produce non-volatile and volatile antifungal antibiotics [45,46]. Furthermore, Palani and Lalithakumari (1999) [47] have demonstrated the efficacy of transgenic strains of this fungal species regarding resistant and sensitive forms of *V. inaequalis* to penconazole, but the role of this fungus may prove to induce tolerance to abiotic stress (salinity) in wheat by improving the antioxidative defense system and gene expression [48].

In addition, certain antagonistic fungi can alter the infectious potential of phytopathogenic organisms by accelerating the decomposition of senescent leaves in combination with antifungal activity; this is the case of *Athelia bombacina* (Link) Pers. and *Coniothyrium* sp. [49] and the yeast *Saccharomyces cerevisiae* (Desm Meyen), which induced the reduction in ascospore production to 95% [50].

Using inverted emulsions containing *T. longibrachiatum* and *C. globosum* has proven their effectiveness against wintering forms of *V. inaequalis*, especially in a formulation containing *C. globosum*. Indeed, Miedtke and Kennel (1990) [33] showed that *C. globosum* is an effective antagonist against the perfect stage of *V. inaequalis* under field conditions. The effectiveness of bio-treatments is much more essential when incorporating senescent leaves to the soil, showing the importance of this cultural practice in optimizing the biological control of apple scab fungus. Carisse and Rolland (2004) [51] have shown that the treatment of apple litter using *Microsphaeropsis ochracea* (Carisse and Bernier) at the right time has negative consequences on the production and ascospore ejection of *V. inaequalis*.

Wound dressings have been considered to be a specific treatment method for wounds affecting woody parts of fruit and forest trees [52,53,54]. We have demonstrated that branch treatment with biogel containing the endophytic yeast *Metschnikowia* sp. led to the healing of the wound caused by the canker more effectively than those treated by tebuconazol. The biological control of tree cankers has been the subject of several studies dealing with the use of microorganisms such as the actinomycete *Saccharothrix yanglingensis* (Yan et al.), sp. nov. against *Valsa mali* (Miyabe and G. Yamada) [55], and antagonistic fungi such as *Trichoderma viride* (Pers.) [56,57] and *T. longibrachiatum* [58].

Biogels have been designed and used to control several fungal plant diseases [59,60,61], but their application could not be more extended as conventional hydrogels were involved in crop protection against water stress by maintaining a good water status of soil [62] for improving germination potential and seedling performance [63] or to control nutrient releasing from fertilizers [64]. Several mechanisms are involved in preventing plant diseases and conferring biocontrol activity to yeasts’ competition for nutrients and space, the secretion of toxins, enzymes, and volatile organic compounds, direct parasitisation, and induction of resistance [65]. More recently, an assessment of the antimicrobial activity of 10 *M. pulcherrima* isolates against 10 key potato phytopathogens by the agar-well diffusion method proved the efficiency of this yeast as a biocontrol agent, revealing biological activity attributed to the enzymes Leucine arylamidase, valine arylamidase, α- and β-glucosidase, and esterases [66]. The in situ experiment demonstrated a reduction by 30%–100% in nine phytopathogens on potato seeds [66]. Some yeasts belonging to the genus *Metschnikowia* were used as biological control agents, mainly against post-harvest diseases of stone and pome fruit, as for *M. pulcherrima* (Pitt and M.W. Mill) [67,68,69] and *M. andauensis* (O. Molnár and Prillinger) [70]. Using a metabolomic analysis of yeast-fungus co-culture, Millan et al. (2022) [71] detected 35 extracellular differential metabolites produced by *Metschnikowia pulcherrima* involved in the biocontrol of gray mold. In this, 3-amino-5-methylhexanoic acid, biphenyl-2,3-diol, and sinapaldehyde were the most active in reducing the infection of tomato and apple fruits up to 90–100%. The same authors showed that these metabolites negatively affected the cell membrane integrity and mycelial morphology of the causal agent (*Botrytis cinerea*) and increased the intracellular level of reactive oxygen species (ROS).

In the present study, we showed that this yeast is useful for protecting apple trees against *Valsa ceratosperma* canker if we include it in a gelled formulation. The progressive evolution of wound healing on trees treated with biogel showed a prolonged efficacy of the bioformulation and probably a covering or colonization of plant parts infected by the pathogen that suggests, in addition to the antibiotic effect, a dressing activity that lasts from 3 to 4 months. In the present work, endophyte yeast *Metschnikowia* sp. has been incorporated into a formulation containing compounds that are not harmful to the environment such as sodium alginate produced by brown algae *Macrocystis pyrifera* (L.) (C. Agardh) and *Laminaria digitata* (Hudson) [72]. This product is characterized by a process known as ionic gelation resulting from the cross-linking network formed from the bonding of Ca^+2^ ions with polyguluronic portions of the polymer units of β(1,4)-D-mannuronic acid and α(1,4)-L-guluronic acid [72]. The alginates not only contribute to the mechanical appearance of the gel but are also considered as a carbohydrate source for the yeast. The gel extracted from the *Linum usitatissimum* seeds was added to improve the mechanical stability of the gel; in fact, the mucilaginous gel of this plant consists essentially of arabinoxylan-type oligosaccharides and pectin [73]. On an experimental scale, the arabinoxylan polymer was used to control the powdery mildew, Rhynchosporiose, and *Ramularia* leaf spots on barley [74].

## 4. Materials and Methods

### 4.1. The Studied Orchards

Two apple orchards were considered for this study that was carried out during the period from December 2020 to May 2021. The first is located in R’Haouat (35°30′09.91″ N, 5°54′21.89″ E, 1400 m) with 226 apple trees (cv. Golden Delicious) spaced 4 m × 4 m, irrigated by the drip system, and fertilized only with organic manure. The chemical treatments were applied to control codling moth (*Cydia pomonella* L.) and mainly scabs and powdery mildew. The second is located in the Bouhmama region, at the southeastern edge of Chelia Mountain (35°18′55.95″ N, 6°42′11.54″ E, 1320 m) with 250 Golden Delicious trees among a total of 460 trees. The irrigation is provided by the drip system and the chemical treatments are mainly applied to control codling moths, mites, and apple scabs. All the trees in both orchards were grafted on MM106 rootstock and planted in 2014.

During the study year, producers have not used pesticides to allow the normal course of the experiment; only the application of bioformulated products that we have designed have been carried out.

### 4.2. Fungal Endophytes

Three endophytic mycotaxa were used as biological substrates for bioformulations: *Trichoderma longibrachiatum* (Rifai) (accession MF144551) and *Chaetomium globosum* (Kunze), both isolated from healthy leaves of native *Hyoscyamus albus* L. (Solanaceae) as well as *Metschnikowia* sp. isolated from the green fruits of *Parthenocissus quinquefolia* (L.) (Planch). Ampelidaceae Fungi are part of a fungal collection established at the plant protection laboratory. The cultural preservation was established according to the standards of the former IMI (International Mycological Institute) [75].

### 4.3. Pathogenic Fungi

The targeted fungi were: *Venturia inaequalis*, the causal agent of apple scab, and *Valsa ceratosperma* inducing branch canker of the apple. The incidence of apple scab and Valsa canker was determined in the year preceding the current study (25.11–14.10% and 29.6–10.4% in R’Haouat and Bouhmama orchards, respectively).

### 4.4. Preparation of the Bioformulations

#### 4.4.1. The Invert Emulsions

They were designed using *T. longibrachiatum* and *C. globosum* as biological fractions. Each bioformulation comprises one aqueous phase and another oily phase. The aqueous phase contains additional conidial suspension of the endophyte (approximately from 106 to 107 spores.mL^−1^) and salicylic acid (0.5 g/500 mL sterilized distilled water), while the oily phase includes a low viscosity vegetable oil to which we have added Tween 80 (0.1%) (3 mL) and sodium alginate (1.1% *w*/*v*) (3 mL) [76]. Both phases were homogenized by mechanical agitation to become yellowish-green (*T. longibrachiatum*) and light-yellow (*C. globosum*) emulsions. The emulsions were preserved in the refrigerator (at from 2° to 4 °C).

#### 4.4.2. The Biogel

A Biogel formulation containing *Metschnikowia* sp. suspension was prepared. The cell suspension of the yeast was prepared (10^7^ cell.mL^−1^) to which we added Tween 80 (0.1%) and then sodium alginate (2% *w*/*v*). The mixture was homogenized by magnetic stirring for 30 min, after which we added oat bran and talc, then the contents we stirred magnetically for one hour. After stirring, the mucilaginous gel extracted from the linseed (*Linum usitatissimum* L.) was added. The resulting gel was kept in the refrigerator (2 °C) for 6 h before applying to the cankers.

### 4.5. Invert Emulsion Application on Wintering Forms of V. inaequalis

The inverted emulsions were applied to the apple senescent leaves (at the litter level) to target the wintering forms of *V. inaequalis*. The choice of this leaf stage is justified by the fact that *V. inaequalis* is a hemibiotrophic fungus that overwinters in senescent and dead leaves, in which microscopic flasked black fruiting bodies (pseudothecia) are developed. These fruiting bodies mature in early spring, ejecting the ascospores in suitable weather conditions. Sub-plots of 4 m^2^ (1 m × 4 m) were selected randomly from each orchard. After repeated cleaning, they were covered with polyester plates for 48 h. Subsequently, senescent leaves were collected and placed on each sub-plot. After 12 h, the leaves were sprayed with emulsions. Each sub-plot is treated with a bioformulation containing a fungal bio-substratum alone. Ten sub-plots have been treated. The sub-plots have been divided into two classes according to a cultural modality; four leaves were left on the soil surface (unincorporated litter), whereas the other four were incorporated into the soil manually (incorporated litter). The remaining two sub-plots (unincorporated and incorporated litter) formed the untreated controls. A second treatment was carried out on the same subplots 15 days after the first, following the same procedures mentioned above.

The sub-plots were covered with a light layer of sterile mesh sleeves and replaced each week by a new tissue; the incubation of the leaves lasted 42 days.

The leaves were sampled and transferred to the laboratory to evaluate the effectiveness of the invert emulsions based on the determination and quantification of ascogenic phases indicating the development and maturity of the wintering pseudothecia of *V. inaequalis*. Thus, the mean number of asci-ejecting ascospores (AA), the mean number of immature asci (IA), and morphologically mature asci (MA) were quantified.

### 4.6. Invert Emulsion Application on Wintering Forms of V. inaequalis

The cankers were treated using the elaborated biogel; 24 wounds (cankers) detected in 14 trees that were treated in the first orchard (R’Haouat) and 21 were detected in 16 trees in the second (Bouhmama). It should be noted that an apple tree may contain more than one canker. The bark surrounding the canker was first removed and then the biogel was applied, forming a gelatinous layer from 5 mm to 12 mm thick, covering the entire wound. The conventional treatment with Folicur^®^ WG (Bayer Crop Science, Leverkusen, Germany) (tebuconazol) at a dose of 40 g/100 L was applied on 10 other cankers, while some cankers (8 for the R’Haouat orchard and 10 for the Bouhmama orchard) did not undergo any treatment.

The treatments were performed on 28 January and 28 February. Careful monitoring of cankers’ evolutions was carried out over 120 days to assess the effectiveness of biogel and Folicur by measuring the wound healing rate. Photos were taken for each canker, first just before treatment and then 30, 60, 90, and 120 days after the last treatment. The resulting photos were then converted to Tiff format and processed via the ImageJ software [77]. First calibrated to a 1 mm unit, the area of the canker (mm^2^) was calculated based on the determination of the depression area that remains in the center of the canker. The canker area was calculated by an algorithm used by the software after spectral scanning.

### 4.7. Statistical Analysis

An analysis of the variance with the LSD Fisher test (*α* = 0.05) was adopted to characterize the effect of the biological substrate (fungal species) of emulsion on the mean number of asci-ejecting ascospores and immature asci, as well as the morphologically mature asci.

## 5. Conclusions

This study showed that the application of bioformulations derived from endophytic fungi can have a positive effect on the phytosanitary status of apple trees and provide natural protection against scab and Valsa canker disease. Inverse emulsion based on *Chaetomium globosum* and *Trichoderma longibrachiatum* shows promise as a biopesticide. We have also shown the importance of cultural practices (the incorporation of litter leaves) in increasing the effectiveness of the formulation, indicating the importance of combining different approaches to apple scab control. Furthermore, using new biogel formulated with the endophytic yeast, *Metschnikowia* sp. offers a new perspective on the natural control of Valsa cankers on apple trees. The use of a biological control involving antagonistic mycoendophytes is much more promising because introduces the conception of suitable formulations without forgetting good cultural practices as part of an integrated disease management system. Based on the results of this study, the bioformulations derived from endophytic fungi based on Chaetomium globosum and Trichoderma longibrachiatum could be recommended in crop protection systems for efficient diseases control, as well as in healthy products.

## Figures and Tables

**Table 1:** Impact of biological treatments of senescent apple leaves with invert emulsions on numbers of asci-ejecting ascospores (AA), immature asci (IA), and morphologically mature asci (MA) of wintering form of *Venturia inaequalis*.

**R’Haouat**						
Unincorporated Leaves *						
	**AA**		**IA**		**MA**	
	Mean number	Evolution	Mean number	Evolution	Mean number	Evolution
Tlong.	8.66 b	↓ 44.84%	32.27 b	↑ 56.88%	18.98 b	↓ 40.68%
Cglob.	4.60 c	↓ 70.70%	41.10 a	↑ 99.81%	7.93 c	↓ 57.21%
Control	15.70 a	-	20.57 c	-	32.00 a	-
Incorporated leaves **						
Tlong.	7,25 b	↓ 39.94%	38.96 b	↑ 125.85%	11.86 b	↓ 43.81%
Cglob.	4.04 c	↓ 66.52%	49.42 a	↑ 186.49%	6.36 c	↓ 69.87%
Control	12.07 a	-	17.25 c	-	21.11 a	-
**Bouhmama**						
Unincorporated leaves *						
	**AA**		**IA**		**MA**	
	Mean number	Evolution	Mean number	Evolution	Mean number	Evolution
Tlong.	6.72 b	↓ 63.19%	36.65 a	↑ 66.36%	15.35 b	↓ 44.56%
Cglob.	3.58 c	↓ 80.39%	39.57 a	↑ 79.61%	9.58 c	↓ 65.40%
Control	18.26 a	-	22.03 b	-	27.69 a	-
Incorporated leaves **						
Tlong.	5.67 b	↓ 57.30%	41.37 b	↑ 164.17%	16.81 a	↓ 9.57%
Cglob.	1.72 c	↓ 87.04%	52.02 a	↑ 232.18%	5.62 b	↓ 69.76%
Control	13.28 a	-	15.66 c	-	18.59 a	-

Tlong. = *Trichoderma longibrachiatum*. Cglob. = *Chaetomium globosum*. Means followed by the same letter(s) within the same column are not significantly different (α = 0.05) according to LSD Fisher test. (*) inspected pseudothecia n = 124 (Tlong.); 120 (Cglob.). (**) n = 137 (Tlong.); 100 (Cglob.).

## Data Availability

Not applicable.
